# A method to assess heart rate variability in neonate rats: validation in normotensive and hypertensive animals

**DOI:** 10.1590/1414-431X20209493

**Published:** 2020-06-26

**Authors:** S.C.F. Freitas, C. Paixão dos Santos, A. Arnold, F.F. Stoyell-Conti, M.R.H. Dutra, M. Veras, M.C. Irigoyen, K. De Angelis

**Affiliations:** 1 Laboratório de Fisiologia Translacional, Universidade Nove de Julho, São Paulo, SP, Brasil; 2 Departamento de Fisiologia, Universidade Federal de São Paulo, São Paulo, SP, Brasil; 3 Unidade de Hipertensão, Instituto do Coração, Faculdade de Medicina de São Paulo, São Paulo, SP, Brasil; 4 DeWitt Daughtry Family Department of Surgery, Miller School of Medicine, University of Miami, Miami, FL, USA; 5 Departamento de Patologia, Faculdade de Medicina, Universidade de São Paulo, São Paulo, SP, Brasil

**Keywords:** Heart rate variability, Neonate rats, Electrocardiogram, Hypertension

## Abstract

Several studies have focused on the heart rate variability (HRV) of murine species, while studies discussing HRV in murine neonates and infants remain scarce, since recording hemodynamic signals through invasive methods in small animals has been found to be quite challenging. Thus, this study aimed at describing and validating a novel method to assess HRV in newborn rats. An electrocardiogram (ECG) system was used to determine RR intervals in awake newborns and evaluate HRV in normotensive (Wistar) and hypertensive (SHR) neonate rats. After birth, ECG was recorded in the awake newborns, and they were allowed to rest on a heated surface, restricted only by the weight of the adhesive ECG electrodes. The electrodes were cut and adapted to provide more comfort to the animal, and gently placed on the newborn's skin. RR intervals were recorded over a 30-min period using an ECG system together with LabChart software (4 KHz). Three sequences of 5 min each from the ECG recording period were analyzed in time and frequency domains, using CardioSeries software. ECG data resulted in a clearly interpretable signal that was used to generate an RR interval sequence through time for the analysis of HRV. SHR neonates presented increased cardiac sympathovagal balance compared to Wistar neonates (low frequency/high frequency: 3.85±0.71 *vs* 0.90±0.09). In conclusion, the ECG setup here described may be used to record RR intervals to assess HRV in neonate rats, thus detecting early impairment of HRV in hypertensive newborns.

## Introduction

When observed on a beat-to-beat- basis, cardiovascular variables exhibit rhythmical fluctuations in their mean values even in the absence of any external stimulus (1). These beat-to-beat variations are the result of autonomic modulation, operating to guarantee a rapid adaptation to environmental or physiological stress (1,2). In this sense, heart rate variability (HRV) is a quantitative marker of autonomic activity in the heart (3).

A number of studies have been devoted to HRV in murine animals, although HRV in neonate and infant rats remains rather understudied. The quality of the signal, the R-to-R intervals (RR) gathered from the electrocardiogram (ECG), and the pulse interval obtained from direct arterial pressure recording are critical components for HRV analysis. In this sense, the acquisition of biological signals in small animals, particularly by invasive arterial pressure procedures, is quite challenging due to the need of specialized surgical skills and expensive recording equipment (4). For this reason, the use of an ECG platform is a viable alternative methodology to obtain RR interval in neonates and assess cardiac autonomic modulation. In this study, we described the use of an ECG setup for the acquisition of RR interval to assess HRV in neonate rats.

Decreased HRV has been found to be a predictor of increased morbimortality in a range of populations, including hypertensives (2,5). Studies with human normotensive offspring of hypertensive parents have demonstrated decreased HRV, characterized by higher cardiac low frequency (LF) band and lower cardiac high frequency (HF) band, together with increased LF/HF ratio (6). This reflects an autonomic nervous system dysfunction, which may be described as a loss of homeostasis between sympathetic and parasympathetic functions (7). Moreover, increased sympathetic activity has been largely observed in SHR rats, at least in the early stage of hypertension (8), and the hypertension is often detected in their 3rd-6th week of life (9,10). The animal also presents reduced parasympathetic activity in adult life (11). Given these findings on HRV impairment in young and adult hypertensives, we may speculate that these unfavorable changes may be manifested in neonate SHRs. Thus, the aim of the present study was to describe and validate a method to assess HRV in awake neonates, comparing normotensive and hypertensive newborns rats.

## Material and Methods

### Animals

Eight-week-old Wistar and SHR rats (3 females and 1 male/each line) were obtained from the Universidade Nove de Julho (UNINOVE) for mating. The procedures and protocols used in this study followed the guidelines of Ethics in Care of Experimental Animals, approved by the Institutional Animal Care and Use Committee, and by UNINOVE's CEUA (AN0011/2017).

After the gestation period, the mothers were isolated in individual boxes and checked daily for birth control. One newborn at a time was separated from the mother during the first 24 h after birth, classified according to sex, and had adhesive electrodes placed on the skin for electrocardiogram (ECG) recording. Ano-genital distance was used for sex identification of neonates. Two to three male neonates of each litter were randomly selected to compose a group (n=8/each group): newborn Wistar control rats (WC neonates) and newborn SHR rats (SHR neonates).

### Setup for RR interval acquisition in neonates: electrodes and ECG system

**Figure 1:**
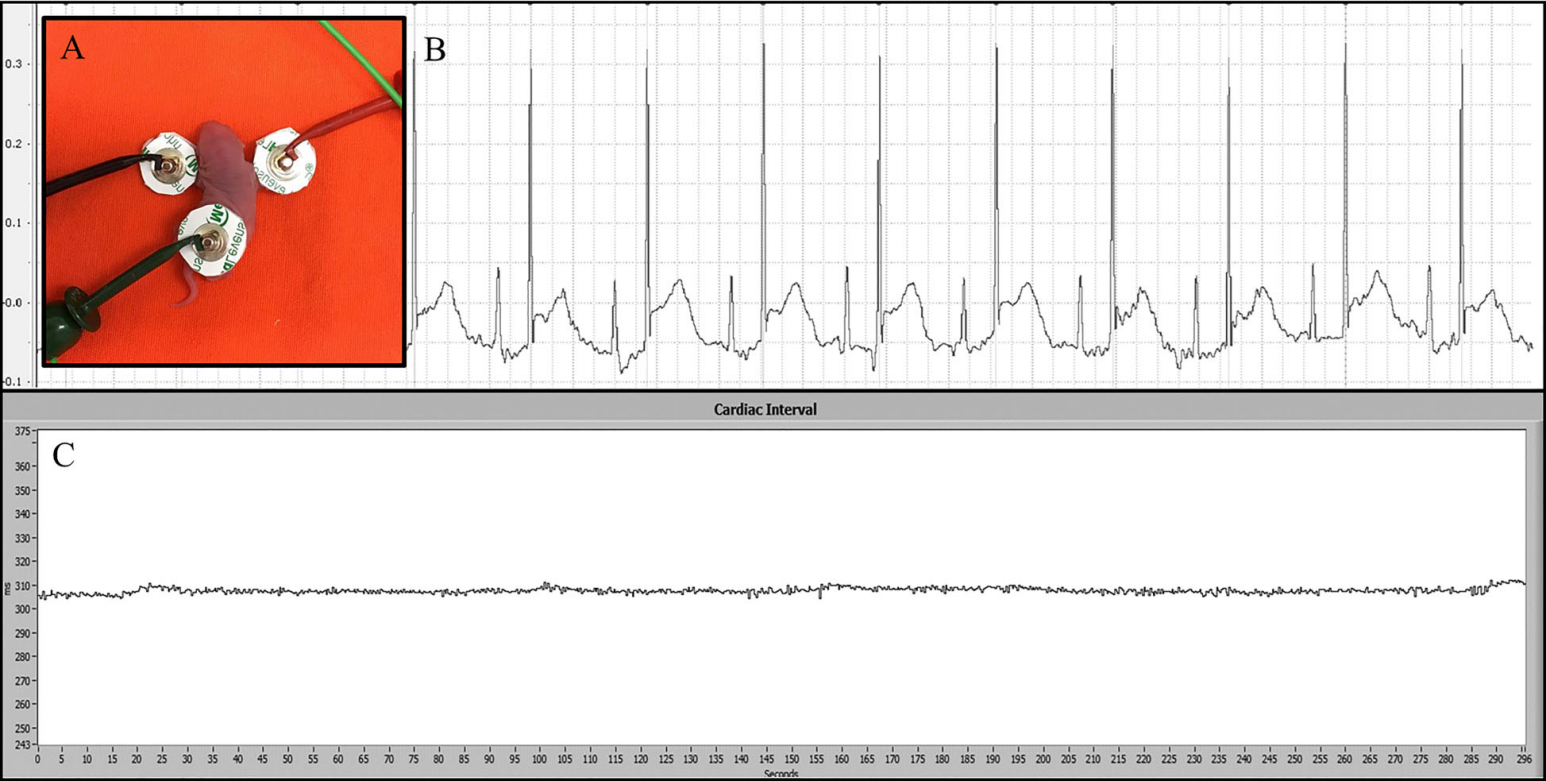
**A**, Electrocardiographic (ECG) data acquisition in a neonate rat. **B**, ECG wave signals. **C**, Tachogram obtained by cardiac interval recording.

On the day of birth, neonates were briefly separated from the mother to record the 30-min period at rest, and then they were euthanized. The animals were kept unsedated and allowed to rest on a heated surface (V831, SonoBel, Brazil), restricted only by the weight of ECG electrodes. Adhesive disposable ECG electrodes (ML02 MedLevensohn, Shanghai INTCO Electrode Manufacturing Co., Ltd., China) were cut to reduce their sizes, removing excess sponge, and were adapted to provide more comfort to the animal, while the part with the silver sensor (Ag/AgCl) was gently placed on the skin of the newborn (Figure 1A). Three Micro-Hook electrodes (1.5 mm Socket, ADInstruments Ltd., New Zealand) were connected to the sensors of the electrodes placed on the right and left limbs of the newborn, as negative and positive poles, respectively, as well as on the back of the animal, as a neutral wire. RR intervals were recorded over a 30-min period using an ECG system (BioAmp FE231, ADInstruments Ltd.) and LabChart software (version 8, ADInstruments Ltd.). The sampling rate was 4000 Hz.

### RR interval detection and HRV analysis

LabChart software detects RR intervals and creates a time-series file containing all RR intervals (tachogram). The software processed the ECG data for beat-to-beat RR detection, using the detection of typical QRS through the specific settings for rat analysis of the LabChart ECG analysis mode.

The entire tachogram was visualized by plotting the RR interval over time through an Excel graphic, and the three most stable sequences of 5 uninterrupted min from the total period were chosen. We chose one sequence at the beginning, one sequence in the middle, and one sequence at the end of the 30 min of ECG recording. The sequences were individually analyzed for HRV and the mean value of the 3 sequences was calculated for each animal. The HRV was analyzed in time and frequency domains (spectral analysis was used for frequency domain parameters) with the CardioSeries (version 2.4, CardioSeries Software, Universidade de São Paulo, Brazil) software, using 512 Fast Fourier Transformation (FFT), interpolation rate of 10 Hz, and the standard frequency-domain for rats: very low frequency band (VLF: 0.00–0.20 Hz), low frequency band (LF: 0.20–0.75 Hz), and high frequency band (HF: 0.75–3.00 Hz). The LF band of RR interval (LF-RR) and the HF band of RR interval (HF-RR) are reported as absolute value and as normalized units (nu).

### Statistical analysis

Data are reported as means±SE. The Kolmogorov-Smirnov test was used to evaluate data normality and *t*-test was used to compare groups. The statistical significance level was established at P≤0.05.

## Results

Figure 1B shows the ECG signal recording on the day of birth from a newborn offspring of a normotensive rat, using the protocol described above. This recording showed clearly interpretable ECG waves, generating an RR interval sequence through time (tachogram, Figure 1C) and making it possible to assess HRV using a specialized software (CardioSeries). This demonstrates the ease of accurately recording RR intervals for HRV assessment in neonates using the protocol proposed in this study.

On the day of birth, SHR neonates presented reduced body mass compared to WC (SHR: 4.25±0.10 *vs* WC: 6.58±0.11 g). The SHR group showed increased cardiac interval compared to WC (SHR: 382±17 ms *vs* WC: 278±5 ms).

**Figure 2:**
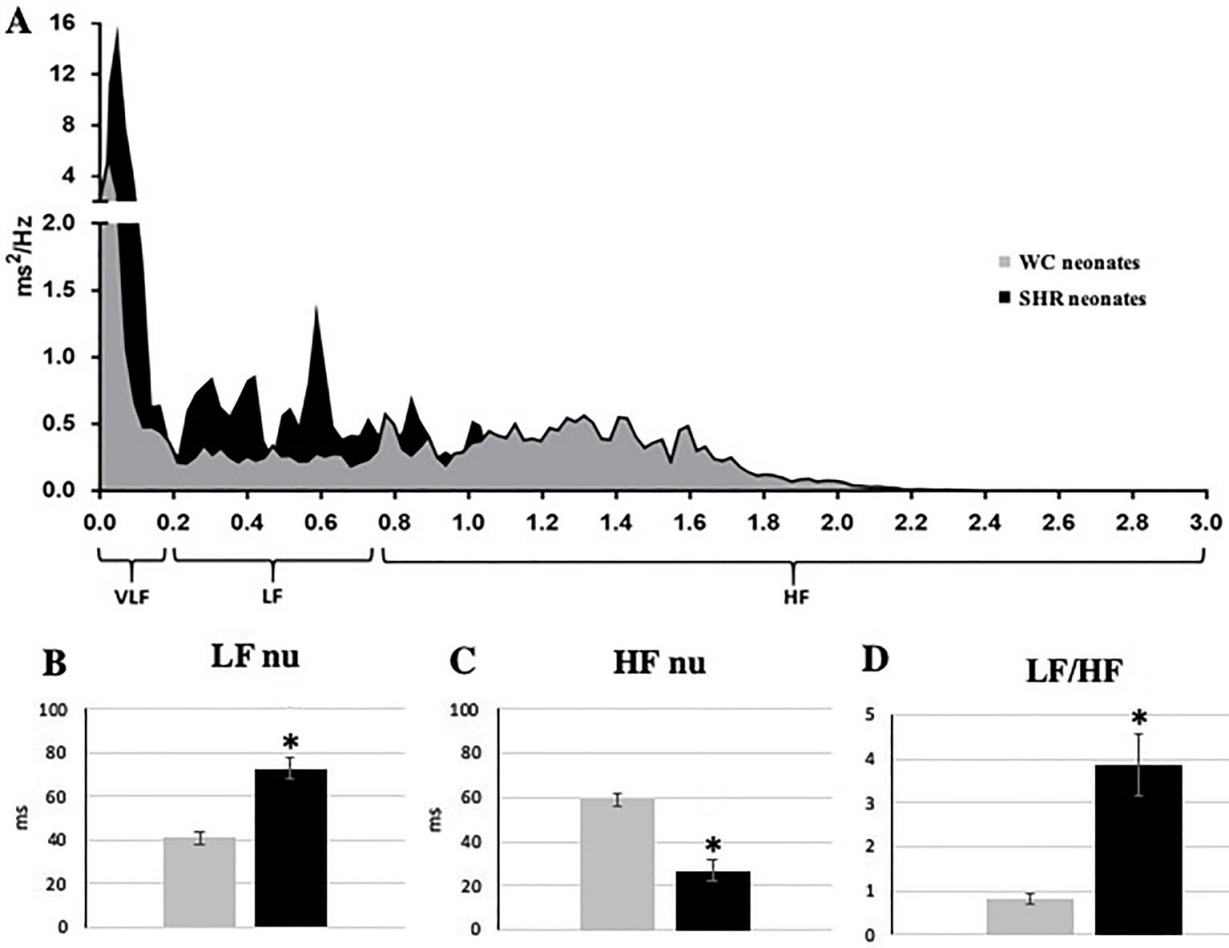
**A**, Power spectrums of Wistar control (WC) and spontaneously hypertensive rat (SHR) neonates. **B,** Low frequency (LF) band of RR interval in normalized units (nu). **C**, High frequency (HF) band of RR interval in nu, **D**, LF/HF ratio. VLF: very low frequency band. Data are reported as means±SE. *P<0.05 *vs* WC neonates (Student’s *t*-test).

Moreover, we validated a method for HRV assessment in neonates, which was demonstrated to be accurate in detecting differences in HRV parameters between normotensive and hypertensive neonate rats. Figure 2A displays the spectrum from a Wistar normotensive neonate and from a hypertensive neonate and shows an increased power in the LF band of the SHR neonate compared to the WC neonate.

There were no differences in total RR variance, standard deviation of RR interval, and root mean square of the successive differences (RMSSD) between groups (Table 1).

**Table 1:** Heart rate variability parameters in time and frequency domains of normotensive Wistar neonates and spontaneously hypertensive (SHR) neonates.

	Wistar neonates	SHR neonates
RR interval total variance (ms^2^)	24.64±3.45	30.24±10.77
RR interval standard deviation (ms)	4.27±0.37	7.19±1.67
RMSSD (ms)	2.02±0.48	2.98±0.59
VLF abs (ms^2^)	1.17±0.44	4.35±0.93*
LF abs (ms^2^)	0.67±0.34	3.16±0.92*
HF abs (ms^2^)	0.73±0.44	0.91±0.28

Data are reported as means±SE (n=8/group). RMSSD: root mean square of the successive differences; VLF: very low frequency band; LF: low frequency band; HF: high frequency band; ms: milliseconds; abs: absolute value. *P<0.05 *vs* Wistar neonates (Student’s *t*-test).

The LF band and VLF band of RR interval were increased in absolute value in SHR neonates compared to WC neonates, although no significant difference was found regarding the HF band in absolute value (Table 1). There was an increase in the LF band and a decrease in the HF band of RR intervals in normalized units in SHR neonates (71.38±4.49 and 28.61±4.49%) compared to WC neonates (43.58±2.25 and 56.41±2.25%) (Figure 2B and C). These alterations in cardiac autonomic balance were clearly demonstrated by the LF/HF ratio, which was significantly higher in the SHR group (WC neonates: 0.90±0.09 and SHR neonates: 3.85±0.71) (Figure 2D).

## Discussion

The aim of this study was to describe and validate a method to assess HRV in newborn rats and for that we had to obtain accurate and reliable ECG setup for the acquisition of RR interval in awake newborns, thus making it possible to evaluate HRV in both normotensive and SHR neonate rats. A significant advantage of this approach is to obtain the RR interval using ECG recordings, a non-invasive approach that was able to analyze the HRV in small rodents (4). Another advantage lies in the quality of the RR interval signal from the ECG, a crucial component for HRV analysis. The ECG platform is considered the “gold standard” for monitoring heart rate (HR) (2), and at a sample rate of 4000 Hz, it provides a clear and easily interpretable signal. Data obtained in this fashion must be viewed with caution, since restraining and anesthesia may affect physiological parameters (4). However, unlike mice with rapid HR (2), neonate rats remain calm and do not need to be sedated or restrained for recordings.

We should bear in mind that the beat-to-beat HR variability assessment reflects the ability of the animal to respond to environmental and physiologic stress, such as changes in volume status, arterial pressure, and autonomic tone (2). The main mechanism underlying HR fluctuations, in short-term cardiovascular control, seems to be the sympathetic and parasympathetic efferent activities modulating the sinus node pacemaker activity (12). This indicates that HRV is dependent on cardiac autonomic regulation (13). In this sense, a decrease in HRV is associated with a decreased ability of an individual to adapt to stress and has been linked to increased mortality in many populations such as myocardial infarcted, hypertensives, and the elderly (2,5). The power spectral analysis of HRV is able to assess sympathetic excitation and concomitant vagal withdrawal, and as such, detect any shift in sympathovagal balance (12). Short recordings of HRV show two primary patterns of oscillation, LF and HF bands (13). Sympathetic modulation may be seen by LF nu or by the LF/HF ratio calculations (12,13). However, the LF and HF absolute values seem to have a major impact on parasympathetic activity, and a large number of studies have found that total vagal blockade is capable of eliminating HF oscillations and reducing the power in the LF absolute value (14). Moreover, since LF nu and HF nu are equal to 100% power, HF nu cannot be seen as an actual representative of parasympathetic activity, as this would require reciprocal changes in sympathetic and parasympathetic modulation, in a very strict complementary interaction (15).

In the literature, SHR rats have been found to present augmented sympathetic activity in the kidney at around 4–5 months old (16) and in the spleen at around 13–20 weeks old (8). In the heart, 45–50-week male SHR rats present higher sympathetic effect and sympathovagal index, followed by reduced parasympathetic effect, compared to control rats (17). However, when the cardiac sympathetic tone of 4–6-month-old SHR rats was evaluated by spectral analysis, no difference was found compared to Wistar rats, while the cardiac parasympathetic tone was reduced in SHR (18). These authors suggest that SHR animals may have an increased sympathetic tone to the heart, leading to hypertension, together with greater parasympathetic control early in life, which may diminish throughout their life span (18). Another study that involved SHR males (11-week-old) reported reduced HRV probably due to intermittent sympathetic or vagal activations or to reduced vagal tone discharge (11).

In the present study, using the described method for HRV assessment in newborn rats, we demonstrated an increase in the index related to cardiac sympathetic modulation in the SHR neonates, as observed by the LF band absolute value and nu, along with LF/HF ratio increase compared to control neonates. Moreover, although RMSSD and HF band in absolute value remained unchanged, the normalized HF band was significantly reduced, while VLF absolute value was increased in SHR compared to control. Recently, associations between VLF and high sympathetic activity have been discussed (19). It has been pointed out that the sympathetic nervous system may play a greater role in the initial and early stages of elevated arterial pressure in both rats and humans with genetic hypertension, with high sympathetic nerve activity present in at least some stages of the diseases (10). A study has found greater levels of catecholamines in the adrenals in SHR pups and higher sympathetic control of the heart through induction of ornithine decarboxylase activity, at 2 days of age (20). Tucker and Johnson (21) have shown that 4-day-old SHR rats implanted with a subcutaneous silver wire electrode present an increased adrenergic contribution to heart rate. In fact, the SHR offspring are normotensive at birth (8,9), similarly to normotensive offspring of hypertensive humans (6), but they inherit impaired autonomic heart modulation, as demonstrated in the present study. They were born with higher cardiac sympathetic modulation and increased LF/HF ratio compared to control neonates.

In conclusion, our findings demonstrated the viability to record RR interval and analyze HRV in newborns using the described ECG setup. Moreover, using this method we were able to demonstrate impaired cardiac autonomic modulation in the SHR neonates since the day they were born, as shown by the higher cardiac sympathetic modulation.
